# Structural, electron transportation and magnetic behavior transition of metastable FeAlO granular films

**DOI:** 10.1038/srep24410

**Published:** 2016-04-14

**Authors:** Guohua Bai, Chen Wu, Jiaying Jin, Mi Yan

**Affiliations:** 1 School of Materials Science and Engineering, State Key Laboratory of Silicon Materials, Key Laboratory of Novel Materials for Information Technology of Zhejiang Province, Zhejiang University, Hangzhou 310027, China

## Abstract

Metal-insulator granular film is technologically important for microwave applications. It has been challenging to obtain simultaneous high electrical resistivity and large saturation magnetization due to the balance of insulating non-magnetic and metallic magnetic components. FeAlO granular films satisfying both requirements have been prepared by pulsed laser deposition. The as-deposited film exhibits a high resistivity of 3700 μΩ∙cm with a negative temperature coefficient despite that Fe content (0.77) exceeds the percolation threshold. This originates from its unique microstructure containing amorphous Fe nanoparticles embedded in Al_2_O_3_ network. By optimizing the annealing conditions, superior electromagnetic properties with enhanced saturation magnetization (>1.05 T), high resistivity (>1200 μΩ∙cm) and broadened Δ*f* (>3.0 GHz) are obtained. Phase separation with Al_2_O_3_ aggregating as inclusions in crystallized Fe(Al) matrix is observed after annealing at 673 K, resulting in a metallic-like resistivity. We provide a feasible way to achieve both high resistivity and large saturation magnetization for the FeAlO films with dominating metallic component and show that the microstructure can be tuned for desirable performance.

Integration of magnetic components, such as inductors, noise suppressors and microwave absorbers on a single chip is critical for the miniaturization of electromagnetic devices and high-speed data transmission^1^,^2^,^3^,^4^,^5^,^6^. Soft magnetic granular films consisting of ferromagnetic metals dispersed in dielectric matrix serve as the potential material for the on-chip electromagnetic applications due to their high electrical resistivity (*ρ*) and large saturation magnetization (*M*_*s*_) compared with traditional ferrites^7^,^8^,^9^,^10^. The metal-insulator films can be divided into three regimes: the metallic, the dielectric and the transition regime depending on the volume fraction (*p*) of the metallic component^8^,^11^,^12^. In the metallic regime where *p* is above the percolation threshold (~0.6), dielectric grains are dispersed in the metallic matrix. Electrons percolate through the interconnected metallic channels, giving rise to a low *ρ* with positive temperature coefficient (TCR). If the film contains excessive dielectric component (*p* < 0.4), it falls in the dielectric regime where metallic grains are embedded in the continuous insulator matrix. In this case, electrons transport via thermally activated tunneling and an insulator-like electrical resistivity with negative TCR is expected. The transition regime from metallic to dielectric is obtained where
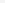
0.4 < *p* < 0.6.

For soft magnetic films used at microwave frequencies, high electrical resistivity is essential to achieve minimum eddy current and impedance matching, where the intrinsic high *ρ* of the dielectric regime is desirable^13^,^14^. Large *M*_*s*_ and broad Δ*f* are also required for device minimization and wide-band applications. It has been challenging to use conventional methods including co-sputtering and reactive sputtering to achieve simultaneous large *M*_*s*_, high *ρ* and broad Δ*f*, which require careful control of the ratio for the metallic and insulating components. The domination of insulator in the dielectric regime significantly decreases the *M*_*s*_ and may even transform the film from ferromagnetic to super paramagnetic^15^,^16^, while a film within the metallic regime cannot satisfy the resistivity requirement^8^. In this work, FeAlO granular films with Fe volume fraction above percolation threshold have been prepared by pulsed laser deposition (PLD) as a non-equilibrium method to achieve large *M*_*s*_, high *ρ* and broad Δ*f*. The as-deposited FeAlO film exhibits a granular structure with Fe nanoparticles dispersed in the Al_2_O_3_ matrix even with a volume fraction of Fe above the percolation threshold (*p* = 0.77). The structural transition upon annealing can be tuned for superior electromagnetic properties with enhanced saturation magnetization, high electrical resistivity and broadened applicable frequency range, which is particularly desirable for microwave absorption.

## Results and Discussion

**Figure 1:**
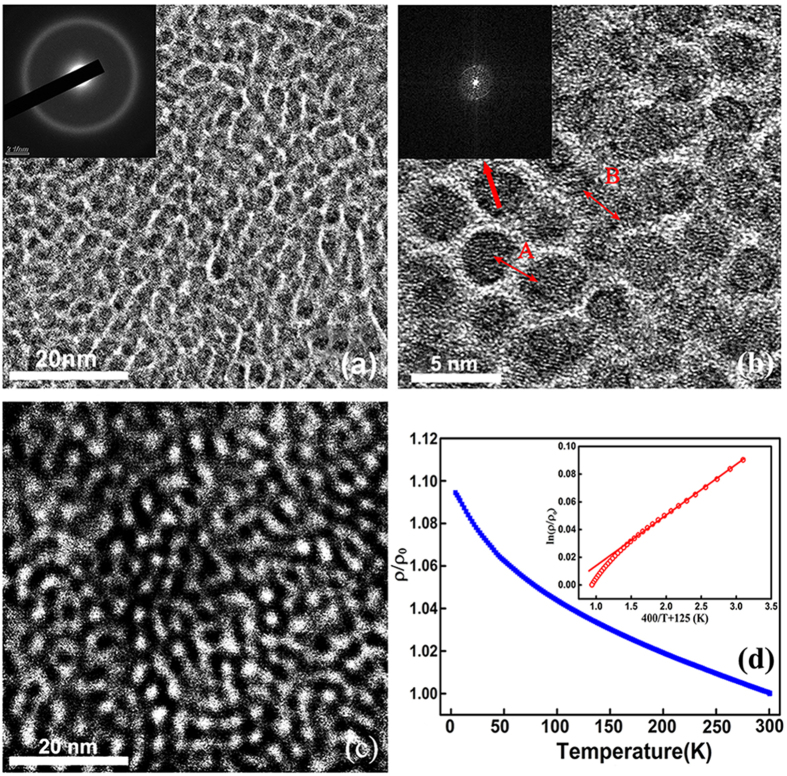
(**a**) TEM image and SAED pattern showing amorphous Fe nanoparticles embedded in the Al_2_O_3_ network for the as-deposited FeAlO film; (**b**) HRTEM image and the FFT pattern as inset taken from the as-prepared film. Site A and site B indicate two types of Fe nanoparticles, isolated by the Al_2_O_3_ network (A) and interconnecting with each other (B); (**c**) The HADDF image of as-deposited FeAlO film. The bright area is attributed to Fe particles and dark area attributed to Al_2_O_3_; (**d**) Temperature dependence of the normalized electrical resistivity for the as-deposited FeAlO film in the range of 4–300 K. The inset shows the fitted ln*ρ* ∼ 400/(T + 125) curve.

FeAlO film prepared by PLD exhibits a granular structure as observed in the TEM images in Fig. 1a,b, where nanoparticles appear as the dark regions isolated by the bright continuum. To reveal the distribution of the different components in the film, high angle annular dark field (HADDF) imaging was performed (Fig. 1c). As the contrast of HADDF image is sensitive to Z^2^ (Z is the atomic number), the bright region and dark region in HADDF image are attributed to Fe and Al_2_O_3_ respectively. The corresponding elemental distribution and chemical state of Fe and Al are also confirmed by EDS mapping and XPS measurements (see Figs S1 and S2 in the Supplementary Information). Consequently, the as-deposited FeAlO film forms with Fe nanoparticles embedded in the Al_2_O_3_ network. The HRTEM image in Fig. 1b presents two types of Fe nanoparticle indicated by A and B. At site A, Fe nanoparticle with a diameter of ~3 nm are completely isolated by the inter-granular Al_2_O_3_ with a thickness of ~0.6 nm. Whereas Fe nanoparticles are interconnected at site B, giving rise to longer pathway (~10 nm) for electron percolation. Note that the interconnected Fe regions are still separated from other Fe nanoparticles by Al_2_O_3_. Selected area electron diffraction (SAED) pattern and Fast Fourier transform (FFT) image as the insets in Fig. 1a,b show that both nanoparticles and the intergranular network are amorphous. The Al_2_O_3_ network accounts for a volume fraction of approx. 23% by measuring the corresponding area in multiple TEM images. Due to the non-equilibrium growth process and the immiscibility of Fe and Al_2_O_3_, the as-deposited FeAlO film contains Al_2_O_3_ precipitating along the Fe particle boundaries despite that the volume fraction of the metallic phase (~0.77) is above the percolation threshold (0.6). The exchange length 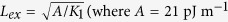 representing the exchange stiffness and *K*_1_ = 48 kJ m^−3^ denoting the magneto crystalline anisotropy^17^) is estimated to be 20 nm for Fe, which is much larger than the Fe particle size and the intergranular distance observed here. Consequently, the Fe particles can be exchange-coupled with each other through the Al_2_O_3_ continuum. The anisotropy field of the FeAlO film is decreased by taking the average of exchange interaction over several nanoparticles, which is beneficial to achieve excellent soft magnetic properties^18^. Previous studies reveal that the electrical resistivity of granular film in dielectric regime follows a negative TCR expressed as *lnρ* *~* *1*/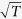 12,19. An additional critical temperature has also been reported corresponding to the transition from superferromagnet (SFM) to superparamagnet (SPM) at low temperature in weak-coupled ferromagnets^20^. In our work, even though the volume fraction of the metallic phase exceeds the percolation threshold, the as-deposited film still exhibits semiconducting behavior with a negative TCR in the temperature range from 4 K to 300 K (Fig. 1d), indicating that the electron mobility increases with temperature and the scattering of electrons from lattice vibration can be neglected. No transition temperature from SFM to SPM is observed here because the *L*_ex_ is much larger than the granular size and intergranular distance. The negative TCR can be fitted with the equation *lnρ* ~ 400/(*T* + 125), as shown in the inset of Fig. 1d. Such conduction behavior can be attributed to fluctuation-induced tunneling which is described as *lnρT*_*1*_/(*T* + *T*_*2*_) in disordered materials characterized by long conducting pathways separated by small insulating barrier^21^. Fitting parameters *T*_*1*_, *T*_*2*_, are related to the tunneling constant *x* and tunneling distant ω by *T*_1_/*T*_2_ = π*x*ω/2. Assuming the localization length 1/*x* to be about 2–3 Å which is the order of the extension of electron wave functions out of metal into vacuum^22^, the tunneling distance can be estimated to be 0.4 ~ 0.6 nm. Such distance is consistent with the thickness of the Al_2_O_3_ layer observed by TEM (Fig. 1b).

**Figure 2:**
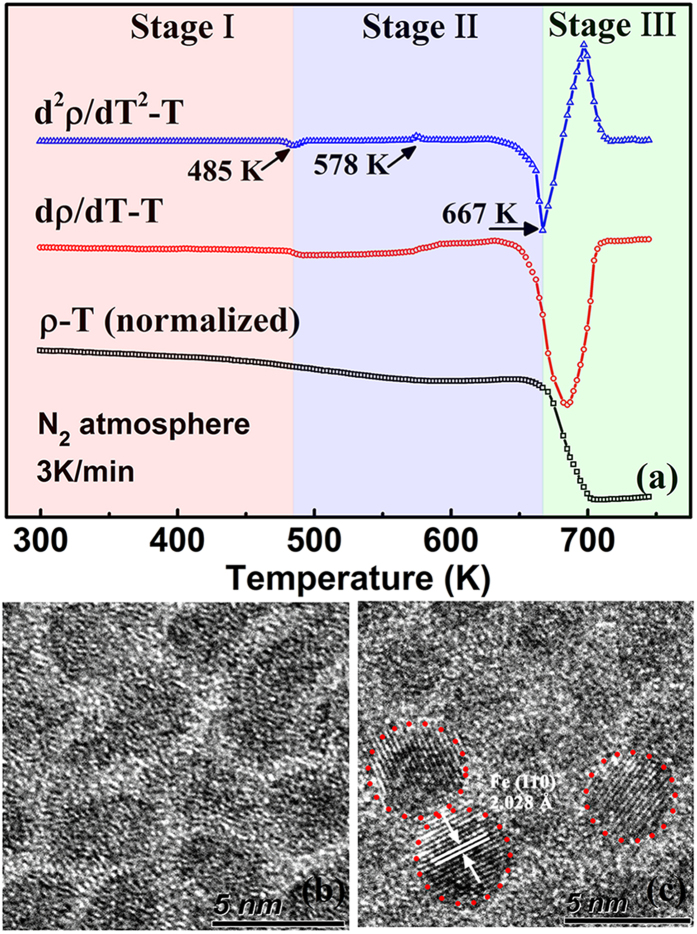
(**a**) Temperature dependence of the normalized electrical resistivity (black) for the as-deposited FeAlO film measured between the range of 300–743 K. *dρ*/*dT* ∼ *T* (red) and *d*^2^*ρ*/*dT*^*2*^ ∼ *T* (blue) curves have also been plotted to identify different stages of the *ρ* ∼ *T* relation; HRTEM images of the FeAlO film after annealing at (**b**) 473 K and **(c)** 573 K for 2 h.

In order to determine thermal stability of the FeAlO film, its electrical resistivity has been measured under N_2_ protection at elevated temperatures with a heating rate of 3 K/min. Figure 2a shows the normalized *ρ*–*T* relation measured in the temperature range of 300–743 K. In order to amplify the change of *ρ*, *dρ*/*dT* and *d*^2^*ρ*/*dt*^2^ have also been plotted as a function of *T*. The films have also been subjected to annealing at 473 K, 573 K, and 673 K for 2 h for TEM investigation to determine the structural transition. The temperature dependence of electrical resistivity can be divided into three stages according to the quadratic differential of *ρ* against *T*. At stage I (T < 485 K), the resistivity decreases slightly as the temperature increasing to 485 K. The HRTEM image of film subjected to 473 K annealing (Fig. 2b) reveals a microstructure similar to the as-deposited FeAlO film with amorphous structure maintained for the Fe nanoparticles embedded in the Al_2_O_3_ network. Consequently, the temperature dependence of resistivity can be explained as discussed for the low temperature region (T < 300 K). At stage 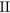 (485 < T < 667 K), the *ρ* experiences faster decreasing as the temperature increases to 578 K. The corresponding HRTEM image of the film annealed at 573 K (Fig. 2c) shows partial crystallization of Fe particles (indicated by the red circle) while the Al_2_O_3_ network is maintained. The relatively faster decrease of the resistivity is attributed to the crystallization of Fe nanoparticles. A slight increase of resistivity between 578 K and 673 K is observed which may be induced by the diffusion of N into the Fe lattice as the measurement is carried out under a nitrogen atmosphere to avoid oxidation. Rapid decline of resistivity takes place at stage III (T > 667 K) with the normalized resistivity *ρ*/*ρ*_0_ falls to nearly 0.1. This is similar to the resistivity of the granular film in the metallic regime.

**Figure 3:**
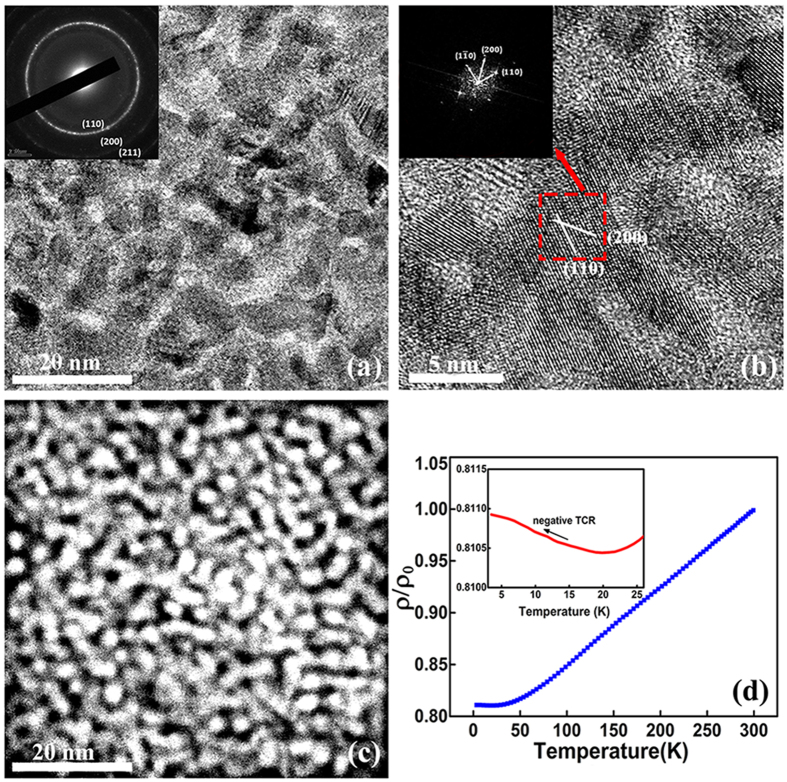
(**a**) TEM image of the film after annealing at 673 K, showing crystallized and interconnected Fe(Al) particles with Al_2_O_3_ as inclusions in the metallic matrix. The insert SAED pattern reveals the (110), (200), (211) crystal planes of the polycrystalline Fe(Al) solid solution; **(b)** HRTEM image of the FeAlO film after annealing at 673 K. The (110) and (200) crystal planes of the Fe(Al) solid solution are identified in the inserted FFT pattern; (**c**) HADDF image of the FeAlO film after annealing at 673 K. The bright Fe grains are more closely packed and connected with each other; (**d**) Temperature dependence of the normalized electrical resistivity for the annealed FeAlO film in the range of 4–300 K. The inset indicates weak electron localization below 20 K.

The drastic change of resistivity in stage III can be interpreted by the distinct structural transition observed by TEM for the film annealed at 673 K as shown in Fig. 3a. The nanoparticles as dark regions in the TEM image become crystallized and interconnect with each other. The corresponding SAED pattern reveals a polycrystalline feature for the film after annealing. Three diffraction rings with d spacings of 2.044 Å, 1.436 Å and 1.176 Å are observed which correspond to the (110), (200), (211) planes of the body-centered cubic (BCC) Fe. The lattice parameters are slightly larger compared to those of the pure Fe (2.026 Å, 1.433 Å and 1.170 Å), indicating that a portion of Al atoms are dissolved into the Fe lattice to form the Fe(Al) solid solution. This is confirmed by the HRTEM and FFT pattern in Fig. 3b. In the HADDF image (Fig. 3c), the Fe nanoparticles interconnect with each other and their contrast becomes brighter due to the coherent scattering of crystallized grains. As the metallic Fe(Al) solid solution and the amorphous Al_2_O_3_ are two immiscible phases, the as-deposited granular structure with Fe(Al) nanoparticles embedded in the Al_2_O_3_ network possesses larger interfacial energy which is metastable. Post annealing promotes the phase separation of Fe(Al) and Al_2_O_3_, reducing the interfacial areas of these two phases via atom diffusion. The crystallized Fe(Al) particles interconnects with each other to form larger size of ~20 nm, while the insulating Al_2_O_3_ becomes isolated and aggregates as inclusions in metallic matrix after annealing. Such drastic structural transition leads to a typical metallic conduction behavior of the film in the temperature range of 4 K–300 K (Fig. 3d). At temperatures above 20 K, the resistivity follows a linear dependence of temperature, which is characterized by electron-phonon scattering of crystal lattice^23^. As shown in Fig. 3a, the Fe(Al) grains are crystallized and interconnected with each other after annealing at 673 K. This structure allows the electrons to percolate through the interconnected Fe(Al) grains. Higher temperature leads to the scattering of more electrons by lattice vibration for a positive TCR. When the temperature is below 20 K, the film exhibit slightly increased resistivity as the temperature is decreased. This behavior can be attributed to electron weak localization effects which stems from the quantum interference of electron mobility when the decoherence length exceeds the grain size at low temperature^19^.

**Figure 4:**
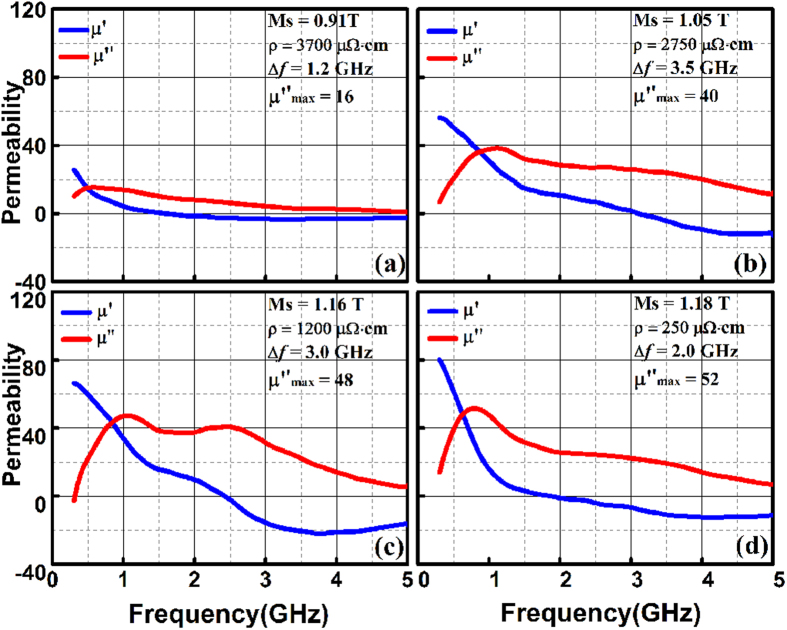
Dynamic and static magnetic properties of the (**a**) as-deposited FeAlO film, and those annealed under different temperatures including (**b**) 473 K, (**c**) 573 K and (**d**) 673 K.

The static and dynamic magnetic properties of the film are also of significant importance for microwave applications. Figure 4 shows the permeability spectra of the FeAlO film annealed under different temperatures. The as-deposited film presents the highest *ρ* of 3700 μΩ∙cm. However, the non-equilibrium PLD process inevitably introduces defects and stress which is the source of magnetic damping and results in a poor frequency response for the as-deposited FeAlO film (Fig. 4a). Post annealing at relatively low temperatures (<473 K) weakens such damping mechanism. Meanwhile, the maintained granular structure results in elevated *M*_*s*_ of 1.05 T, 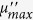
of 40 and a resistivity of 2750 μΩ∙cm (Fig. 4b). The *M*_*s*_ and the 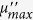 can be further improved to 1.16 T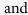 48 after annealing at 573 K when the Fe particles is partially crystallized with the Al_2_O_3_ network preserved for high electrical resistivity (1200 μΩ∙cm). The coexistence of amorphous and crystallized Fe(Al) particle gives rise to double resonance peaks for broadened FWHM Δ*f* up to 3.0 GHz in the permeability spectra (Fig. 4c). The imaginary permeability remains around 40 in the range from 0.7 GHz to 2.5 GHz, indicating steady microwave absorption behavior within such broad frequency band. The formation of metallic continuum after annealing at 673 K allows percolation conduction across the Fe(Al) particles, leading to decreased electrical resistivity (250 μΩ∙cm) with deteriorated high frequency response of the film (Fig. 4d). Table 1 summarizes the electrical and magnetic properties of the FeAlO films compared with those of other granular films. The electrical resistivity of the FeAlO films is as high as above 1200 μΩ∙cm. The *f*_*r*_ and *M*_*s*_ are both adjustable through post annealing. Optimized value of Δ*f* (above 3 GHz) can be obtained after annealing at 473 K and 573 K. The FeAlO films reported here exhibit excellent comprehensive performance, which serve as promising candidates for microwave absorption^29^.

## Conclusion

In summary, metastable FeAlO granular film which falls into the metallic regime has been fabricated by PLD as a non-equilibrium method to achieve both large *M*_*s*_ and high *ρ*. The granular structure can be modulated by post annealing at different temperatures, leading to excellent microwave absorption performance with enhanced *M*_*s*_ (>1.05 T), high *ρ* (>1200 μΩ∙cm) and broadened Δ*f* (>3.0 GHz). The structural transition upon annealing of the metastable FeAlO granular film reveals a feasible way to understand and tune the performance of granular films.

## Methods

### Samples

FeAlO films were fabricated on Si (100) single crystal substrates by PLD. A Fe_0.8_Al_0.2_ alloy disk with a diameter of 1 inch was used as the target. A static magnetic field of 250 Oe was applied parallel to the substrate surface during deposition to induce in-plane uniaxial magnetic anisotropy in the film. The deposition chamber was evacuated to a base pressure of 6 × 10^−6^ torr. The wavelength of the laser is 248 nm. The incident laser energy is 250 mJ with a repetition of 10 Hz and a deposition time of 4.5 h. The FeAlO films have been grown repeatedly and similar granular structure has been obtained which confirms the reproducibility. The as-deposited sample was cut into four pieces, three of which were subjected to vacuum annealing at temperatures ranging from 473 K to 773 K for 2 h. The film was also deposited on a NaCl substrate from which the film can be separated for transmission electron microscopy (TEM) investigation by dissolving the substrate in deionized water.

### Measurements

The microstructure of the film was examined by a high resolution TEM (JEM 2100 F) with the film deposited on NaCl substrate. HADDF images and EDS mapping were also taken using the STEM mode. A superconducting quantum interference device (SQUID) was used to measure the static magnetic properties of the films. The room temperature resistivity was measured by four-point method (RTS-8). The chemical state of the film was investigated with XPS (Escalab 250Xi). The high temperature dependence of resistivity of the sample was obtained using four-point method under N_2_ atmosphere (DZL-100). The low temperature dependence of resistivity was measured by Physical Property Measurement System (PPMS). The permeability spectra were measured by shorted microstrip transmission-line perturbation method with a vector network analyzer (VNA, Agilent E8363B)^30^.

## Additional Information

**How to cite this article**: Bai, G. *et al.* Structural, electron transportation and magnetic behavior transition of metastable FeAlO granular films. *Sci. Rep.* **6**, 24410; doi: 10.1038/srep24410 (2016).

## Supplementary Material

Supplementary Information

## Figures and Tables

**Table 1:** Magnetic performance and electrical resistivity of the FeAlO films compared with other granular films reported in the literature.

Fabrication method	Composition	*M*_s_ (T)	ρ (μΩ∙cm)	*f*_*r*_ (GHz)	Δ*f* (GHz)
Pulsed laser deposition	FeAlO 473 K HT	1.05	2750	1.1	3.5
	FeAlO 573 K HT	1.16	1200	1.1 and 2.5	3.0
Reactive sputtering	FeAlO^24^	1.8	66	0.8	–
Reactive sputtering	CoAlO^25^	0.95	1000	2.45	–
Reactive sputtering	FeCoAlO^26^	1.6	400	2.3	1.0
Reactive sputtering	FeCoAlN^27^	1.08	275	1.72	1.5
Co-sputtering	CoZnO^28^	1.47	190	3.78	1.0
Reactive sputtering	FeCoSiN^7^	1.0	1000	1.1	0.4
Reactive sputtering	FeN^29^	1.26	190	1.1-2.2	1.7
