# Supplementary material for: Structural, electron transportation and magnetic behavior transition of metastable FeAlO granular films
Source: Sci Rep. 2016 Apr 14;6:24410. doi: 10.1038/srep24410 (PMC4830969; doi:10.1038/srep24410)
Supplement: Supplementary Information [file srep24410-s1.pdf]

# Supplementary Information

# Structural, electron transportation and magnetic behavior transition of metastable FeAlO granular films

Guohua Bai, Chen Wu*, Jiaying Jin & Mi Yan*

*School of Materials Science and Engineering, State Key Laboratory of Silicon Materials, Key Laboratory of Novel Materials for Information Technology of Zhejiang Province, Zhejiang University, Hangzhou 310027, China*

In this supplementary material, we provide further details on the spatial distribution of different components (Figure S1) and the chemical state (Figure S2) of the as-deposited FeAlO film.

**Elemental mapping of the as-deposited FeAlO film**

For the as-deposited FeAlO film, a granular structure has been observed with TEM. HADDF imaging was also used to show the spatial distribution of Fe and $Al_2O_3$. To provide further elemental distribution of Fe, Al and O, we performed HADDF-STEM-EDS mapping. In the HADDF image, the bright and dark regions correspond to the Fe and $Al_2O_3$ respectively since the contrast of HADDF image is sensitive to $Z^2$(Z is the atomic number). The elemental mapping shows that the Fe aggregates as particles while Al and O are distributed as the network isolating Fe particles. Combined elemental mapping with TEM and HADDF observations confirms the granular structure of as-deposited FeAlO film with Fe nanoparticles embedded in the $Al_2O_3$ network.

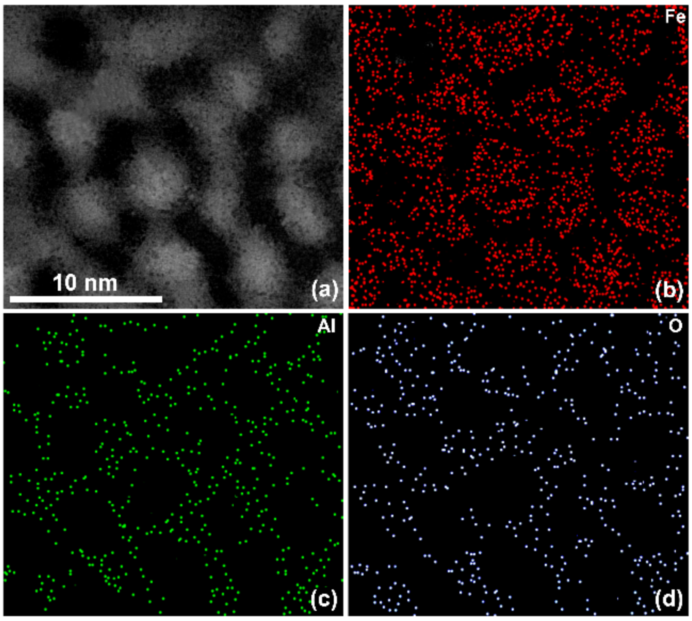


**Figure S1.** HADDF-STEM-EDS mapping to illustrate the elemental distribution of (b) Fe, (c) Al and (d) O for the as-deposited FeAlO film. The Fe forms nanoparticles which are isolated by the $Al_2O_3$ network

**Chemical state of the as-deposited FeAlO film**

To further confirm the chemical state of Fe and Al, XPS measurement has been carried out. The sample was transferred to the XPS chamber immediately after taking out of the PLD chamber to avoid any possible oxidation. The two peaks at ~706.8 eV and ~720.2 eV in the Fe spectrum **(a)** correspond to the Fe $2p_{3/2}$ (metal) and Fe $2p_{1/2}$ (metal)[1], indicating that the Fe exists in the metallic state for the nanoparticles. The Al 2p peak is located at 74.1 eV in the XPS spectrum in **(b)**, which is about 1.3 eV larger than that of the metallic Al[1], suggesting oxidation of Al in the as-deposited film.

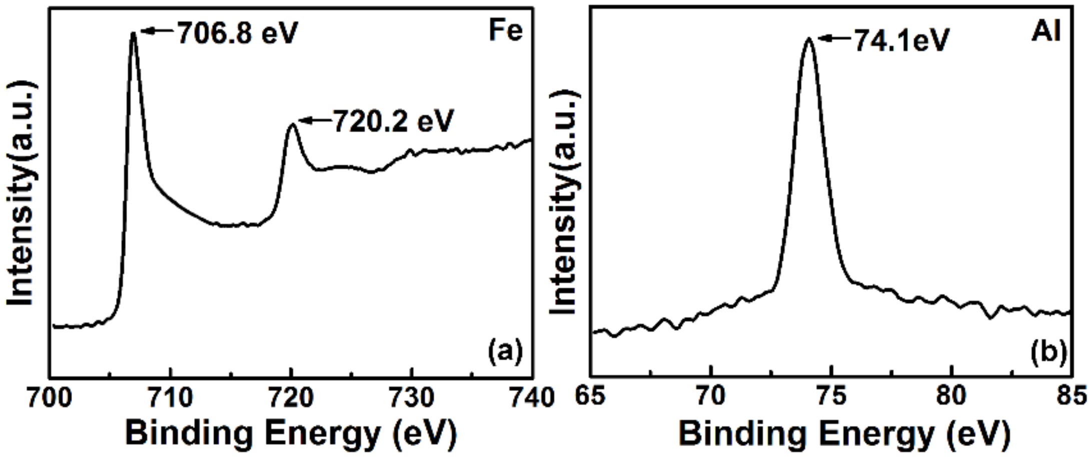


**Figure S2.** (a) Fe and (b) Al spectra taken from the as-deposited FeAlO film. The two peaks at ~706.8 eV and ~720.2 eV in the Fe spectrum belong to Fe $2p_{3/2}$ (metal) and Fe $2p_{1/2}$ (metal) respectively, suggesting that the Fe nanoparticles exist in the metallic state. The Al 2p peak is located at 74.1 eV in the XPS spectrum, corresponding to oxidized Al in the as-deposited film.

1. Powell, C. J. Recommended Auger parameters for 42 elemental solids. *J. Electron Spectros. Relat. Phenomena* **185**, 1–3 (2012).
